# Outcomes for a digital prescription mobile application for adults with irritable bowel syndrome: an uncontrolled trial

**DOI:** 10.1186/s12876-026-04964-6

**Published:** 2026-06-11

**Authors:** Mohsin F. Butt, Maiss Reghefaoui, Debbie Bush, Abdulsalam Aliyu, Ana-Maria Darie, Aleesha Mohanan, Alice Sibelli, Timothy R. Card, Maura Corsetti

**Affiliations:** 1 NIHR Nottingham Biomedical Research Centre, Nottingham University Hospitals NHS Trust and the University of Nottingham, Nottingham, United Kingdom; 2 Nottingham Digestive Diseases Centre, University of Nottingham, Queen’s Medical Centre, Nottingham, NG7 2UH United Kingdom; 3 Wingate Institute of Neurogastroenterology, Centre for Neuroscience, Trauma and Surgery, Blizard Institute, Barts and The London School of Medicine and Dentistry, Queen Mary University of London, London, E1 2AJ United Kingdom

**Keywords:** Abdominal pain, Psychological therapy, Digital therapeutics, Health outcomes, Digital prescription

## Abstract

**Background:**

Behavioral therapies are effective in the management of irritable bowel syndrome (IBS). Mahana™ IBS is a mobile phone application designed to deliver 10 sessions (10-12 weeks) of gut-directed cognitive behavioral therapy (CBT) to adults with IBS.

**Objectives:**

To evaluate patient compliance with the Mahana™ mobile phone CBT application and the change in gastrointestinal and psychological symptoms in a real-world UK setting.

**Methods:**

This single-center prospective study recruited adults with Rome IV IBS from a tertiary care outpatient setting. At baseline and 12-week follow-up, patients completed questionnaires assessing gastrointestinal symptoms (IBS symptom severity scale [IBS-SSS]), depression (patient health questionnaire-9 [PHQ-9]), and anxiety (generalized anxiety disorder-7 [GAD-7]).

**Results:**

Thirty patients (20/30 female, mean [SD] age 44.57 [14.70]) consented to treatment and completed pre-intervention questionnaires, among whom 20 (66.7%) provided follow-up questionnaire data. Among the 20 individuals with two-point data, there was a statistically significant reduction in the IBS-SSS (P<0.001), GAD-7 (P=0.02), and PHQ-9 (P=0.04) scores. Fourteen (70%) patients reported a clinically significant improvement (≥50 point reduction) in the IBS-SSS; these individuals tended to be older (P=0.04) and male (P=0.04). Patients with IBS-diarrhea reported the greatest improvement in the IBS-SSS versus those with IBS-constipation and IBS-mixed subtypes. The baseline IBS-SSS (P=0.35), GAD-7 (P=0.19) and PHQ-9 (P=0.19) scores were not significantly different between persons who completed the entire CBT intervention versus those who did not.

**Conclusion:**

This study provides preliminary evidence that the Mahana™ IBS CBT application is associated with a statistically significant improvement in gastrointestinal and psychological symptoms in a real-world UK clinical setting.

**Trial registration:**

This trial was registered on ClinicalTrials.gov (trial registration number: NCT07008404) on 6 June 2025

**Supplementary Information:**

The online version contains supplementary material available at 10.1186/s12876-026-04964-6.

## Key summary statement

### What is already known on this topic

- Irritable bowel syndrome (IBS) is a disorder of gut–brain interaction that can respond to gut-directed behavioral therapy, such as cognitive behavioral therapy (CBT).
- Widespread adoption of gut-directed CBT remains limited due to challenges with clinician availability and delivery infrastructure, and these can be addressed using digital platforms.

### What this study adds

- This prospective evaluation (NCT07008404) represents the first published real-world clinical study of the Mahana™ IBS application – a digital gut-directed CBT mobile phone application for the treatment of IBS – in the United Kingdom.
- Mahana™ IBS is associated with clinically meaningful improvements in gastrointestinal symptoms, anxiety, and depression within routine clinical practice in the National Health Service.

### How this study might affect research, practice or policy

- This pilot study supports the development of larger, controlled trials to evaluate the effectiveness and scalability of digital CBT for people living with IBS.

## Introduction

Irritable bowel syndrome (IBS), which affects approximately 4% of the UK population [1, 2], is a disorder of gut-brain interaction (DGBI) characterized by altered bowel movements (either constipation, diarrhea, or both) and abdominal pain related to defecation [3, 4]. The etiology of IBS is best conceptualized through the biopsychosocial model, which addresses the inter-relationship between psychosocial (e.g., psychological state and social support) and physiological (e.g., disturbances in gastrointestinal motility, visceral hypersensitivity, and altered central nervous system processing) factors [5]. 

As the etiology of IBS is multifactorial and incompletely understood [6], effective evidence-based treatments are limited [7]. Suboptimal management may impair quality of life [8], work and social functioning [9, 10], and psychological well-being [11]. IBS and common psychological disorders (i.e., anxiety and depression) frequently co-occur [12, 13] and there is a bidirectional relationship between both elements [14, 15]. Gastrointestinal-focused psychological therapies, such as cognitive behavioral therapy (CBT) and hypnotherapy, are effective in the management of IBS [16] and are recommended by the UK National Institute of Clinical Excellence for the treatment of individuals with refractory IBS i.e., those with ongoing symptoms after 12 months despite being offered appropriate medications and lifestyle advice [17].

Supported by at least 20 published randomized controlled trials, CBT has the greatest evidence base among the various forms of behavioral therapy for the treatment of IBS [18]. CBT focuses on unlearning maladaptive coping skills that have developed as a response to gastrointestinal symptoms and/or stress [19]. CBT may be delivered in various formats, including digital platforms and traditional face-to-face settings, yet access remains suboptimal worldwide [20]. In resource-limited healthcare systems, digitally delivered CBT may improve access to psychological therapy by offering flexible, self-paced engagement at the participant’s convenience, while eliminating the need for travel and associated expenses. Mahana™ IBS is a UK Conformity Assessed, Conformité Européenne, and Food and Drug Administration-cleared prescription mobile application designed to deliver 10-12 weeks (10 sessions) of gut-directed CBT to adults with IBS (Supplementary Table 1). Mahana™ IBS does not require therapist support and has been developed from a clinically validated web-based CBT program, Regul8 [21, 22]. 

Although Mahana™ IBS has been tested in North America among patients with moderate symptom severity of IBS [23], it remains unclear whether its efficacy differs across populations worldwide or is influenced by baseline IBS symptom severity. Indeed, evidence from CBT more broadly suggests that treatment response may vary according to cultural context, healthcare setting, and baseline psychological comorbidity, underscoring the need for evaluation in diverse cohorts [24, 25].

This prospective study evaluated patient compliance with the Mahana™ application and the change in gastrointestinal and psychological symptoms in a real-world UK setting.

## Methodology

### Study aims

The primary aim of this study was to assess the change in the IBS symptom severity scale (IBS-SSS), patient health questionnaire-9 (PHQ-9; a measure of depressive symptoms), and generalized anxiety disorder-7 (GAD-7; a measure of anxiety) scores between baseline and after completion of the Mahana™ IBS application. 

The secondary aims were to identify the baseline characteristics that were associated with improvement in the IBS-SSS, PHQ-9, and GAD-7, and factors that were associated with poor compliance with the application.

### Study design and participants

This single-center prospective study was conducted in a tertiary care neurogastroenterology outpatient clinic between 1st January 2024 and 28th February 2025. Adults (aged ≥ 18 years) meeting Rome IV criteria for IBS [26] were referred by an attending/consultant gastroenterologist (MC) to the Mahana™ CBT application in addition to standard medical management in accordance with British Society of Gastroenterology guidelines [27]. Patients with prior exposure to gut-related psychological interventions were not enrolled. Recruitment was discontinued prior to reaching the target sample size following the sponsor’s decision to cease further development and commercialization of the application for strategic reasons unrelated to safety or efficacy signals observed in this study. Data have previously been published in abstract form [28].

### Instruments

At baseline and 12-week follow-up, patients completed the IBS-SSS [29], PHQ-9 [30], and GAD-7 [31] scales to measure severity of IBS symptoms, depression, and anxiety, respectively. Patients were not re-contacted during the 12-week study period, and outcome questionnaires were administered face-to-face by a research coordinator (DB).

The IBS-SSS is a composite measure that captures multiple dimensions of IBS, including abdominal pain, abdominal distension, satisfaction with bowel habits, and the impact of symptoms on quality of life. Each item is scored on a scale from 0 to 100, yielding a total score ranging from 0 to 500, with higher scores indicating greater symptom severity. Mild, moderate, and severe cases of IBS were indicated by scores of 75 to 175, 175 to 300 and >300, respectively. A clinically significant improvement in the IBS-SSS is defined as a 50 point or greater reduction in symptom severity at follow-up versus baseline [29]. 

The PHQ-9 is a 9-item self-report measure assessing depressive symptoms over the past two weeks. Items are scored from 0 to 3, yielding a total score from 0 to 27, with higher scores indicating greater severity. Severity is categorized as minimal (1 to 4), mild (5 to 9), moderate (10 to 14), moderately severe (15 to 19), and severe depression (20 to 27).

The GAD-7 is a 7-item self-report questionnaire used to assess the severity of generalized anxiety symptoms over the past two weeks. Each item is scored from 0 (not at all) to 3 (nearly every day), producing a total score ranging from 0 to 21. Severity is categorized as minimal (0 to 4), mild (5 to 9), moderate (10 to 14), and severe anxiety (15 to 21).

### Statistical analysis

The Wilcoxon signed-rank test was used to evaluate the within-group change in questionnaire scores (IBS-SSS, PHQ-9, and GAD-7) between baseline and follow-up. Matched samples, unmatched samples, and correlations were analyzed using the Wilcoxon signed-rank test, the Mann–Whitney U test, and Spearman’s ρ, respectively, to determine baseline characteristics associated with clinical response to the application and poor compliance. Patients who did not comply, or “drop-outs”, were defined as persons who (i) did not complete all ten CBT modules or (ii) failed to complete the follow-up questionnaires. Data were analyzed using SPSSV29.0 and *P* values < 0.05 were considered statistically significant. When comparing characteristics pairwise between three groups (e.g., between IBS subtypes), the significance threshold was adjusted to 0.017 using the Bonferroni correction.

#### Clinical trial registration and ethical approval

This study was registered on ClinicalTrials.gov (trial registration number: NCT07008404) on 6 June 2025. The study was approved by the Research Ethics Committee at Nottingham University Hospitals NHS Trust (REC approval number: 23/SS/0091). All participants provided written informed consent prior to participation.

## Results

### Sample characteristics

**Fig. 1:**
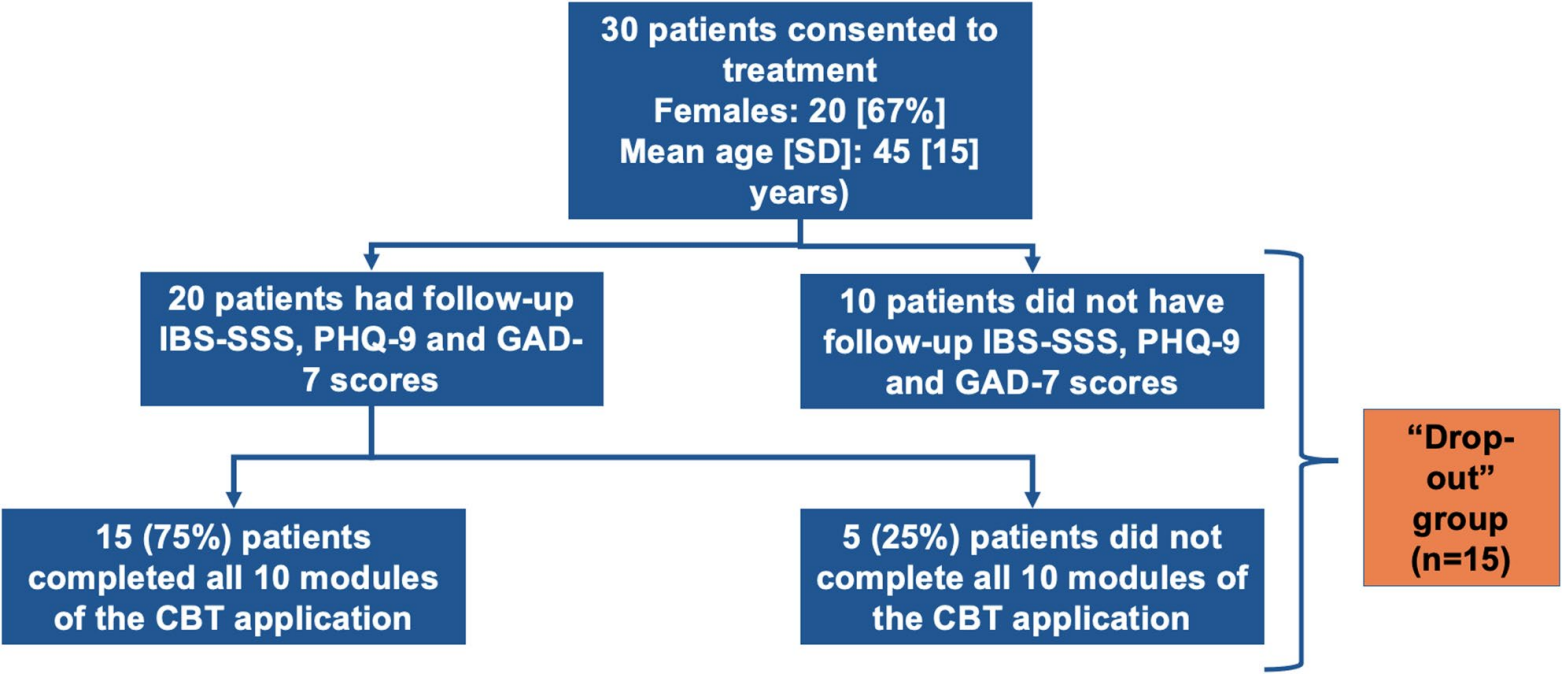
Study Flowchart. Abbreviations: CBT, cognitive behavioral therapy; GAD-7, generalized anxiety disorder-7; IBS-SSS, irritable bowel syndrome symptom severity scale; PHQ-9, patient health questionnaire-9

Thirty patients consented to treatment and completed pre-intervention questionnaires **(**Table 1**)**, among whom 20 (66.7%) provided follow-up questionnaire data. Eleven (55%), five (25%), and four (20%) patients had respectively IBS-constipation (IBS-C), IBS-mixed (IBS-M), and IBS-diarrhea (IBS-D). Among the 20 patients, 15 (75%) completed the entire CBT course **(**Fig. 1**).** The five patients who did not complete the entire CBT intervention along with the 10 patients who did not provide follow-up data constituted the “drop-out” group (*n* = 15) **(**Fig. 1**).**

**Table 1:** Baseline demographic, clinical, and resource-use characteristics

Subgroup Labels	Patients who consented for treatment (*n* = 30)	Patients with two-point data *AND* completed the entire CBT course (*n* = 15)	Patients with two-point data (*n* = 20)	Patients with no follow-up data *OR* partially completed the CBT course (*n* = 15)	Patients with no follow-up questionnaire data (*n* = 10)	Patients with follow-up data who partially completed the CBT course (*n* = 5)	*P* value^a^ A vs. C	*P* value ^a^ A vs. D	*P* value ^a^ A vs. E	*P* value ^a^ B vs. D
		Subgroup A	Subgroup B	Subgroup C	Subgroup D	Subgroup E				
Demographics										
Age, mean (SD)	44.57 (14.70)	48.13 (13.98)	46.30 (15.33)	41.00 (14.99)	41.10 (13.41)	40.80 (19.54)	0.22	0.26	0.45	0.40
Sex, female, *n* (%)	20 (66.67)	9 (60.00)	13 (65.00)	13 (86.67)	9 (90.00)	4 (80.00)	0.22	0.18	0.61	0.21
Sex, male, *n* (%)	8 (26.67)	6 (40.00)	7 (35.00)	2 (13.33)	1 (10.00)	1 (20.00)	0.22	0.18	0.61	0.21
BMI, mean (SD)	26.35 (7.25)	29.61 (7.97)	27.84 (7.70)	23.09 (4.74)	23.36 (5.41)	22.54 (3.50)	0.006	0.03	0.03	0.07
IBS subtype										
Diarrhea, *n* (%)	4 (13.33)	4 (26.67)	4 (20.00)	0 (0.00)	0 (0.00)	0 (0.00)	0.10	0.13	0.53	0.27
Constipation, *n* (%)	14 (46.67)	8 (53.33)	11 (55.00)	7 (46.67)	4 (40.00)	3 (60.00)	> 0.99	0.13	> 0.99	0.70
Mixed, *n* (%)	10 (33.33)	3 (20.00)	5 (25.00)	8 (53.33)	6 (60.00)	2 (40.00)	0.13	0.09	0.56	0.11
Previous abdominal surgery, *n* (%)	6 (20.00)	4 (26.67)	4 (20.00)	3 (20.00)	3 (30.00)	5 (100.00)	> 0.99	> 0.99	0.53	0.66
Time duration (months) before symptom onset and CBT application delivery, mean (SD)	291.68 (561.48)	502.40 (797.20)	408.50 (683.98)	116.1 (88.81)	87.25 (43.53)	173.75 (133.60)	0.54	0.46	0.95	0.37
Resource use, mean (SD)^b^	2.50 (1.11)	2.67 (1.29)	2.40 (1.23)	2.33 (0.90)	2.70 (0.82)	1.60 (0.55)	0.39	0.98	0.07	0.48

*Abbreviations*: *BMI* body mass index, *CBT* cognitive behavioral therapy

^a^ Wilcoxon-Rank Sum or Fishers-Exact test for continuous and categorical variables, respectively

^b^ Sum of the number of investigations and medications

### Change in questionnaire scores 

**Fig. 2:**
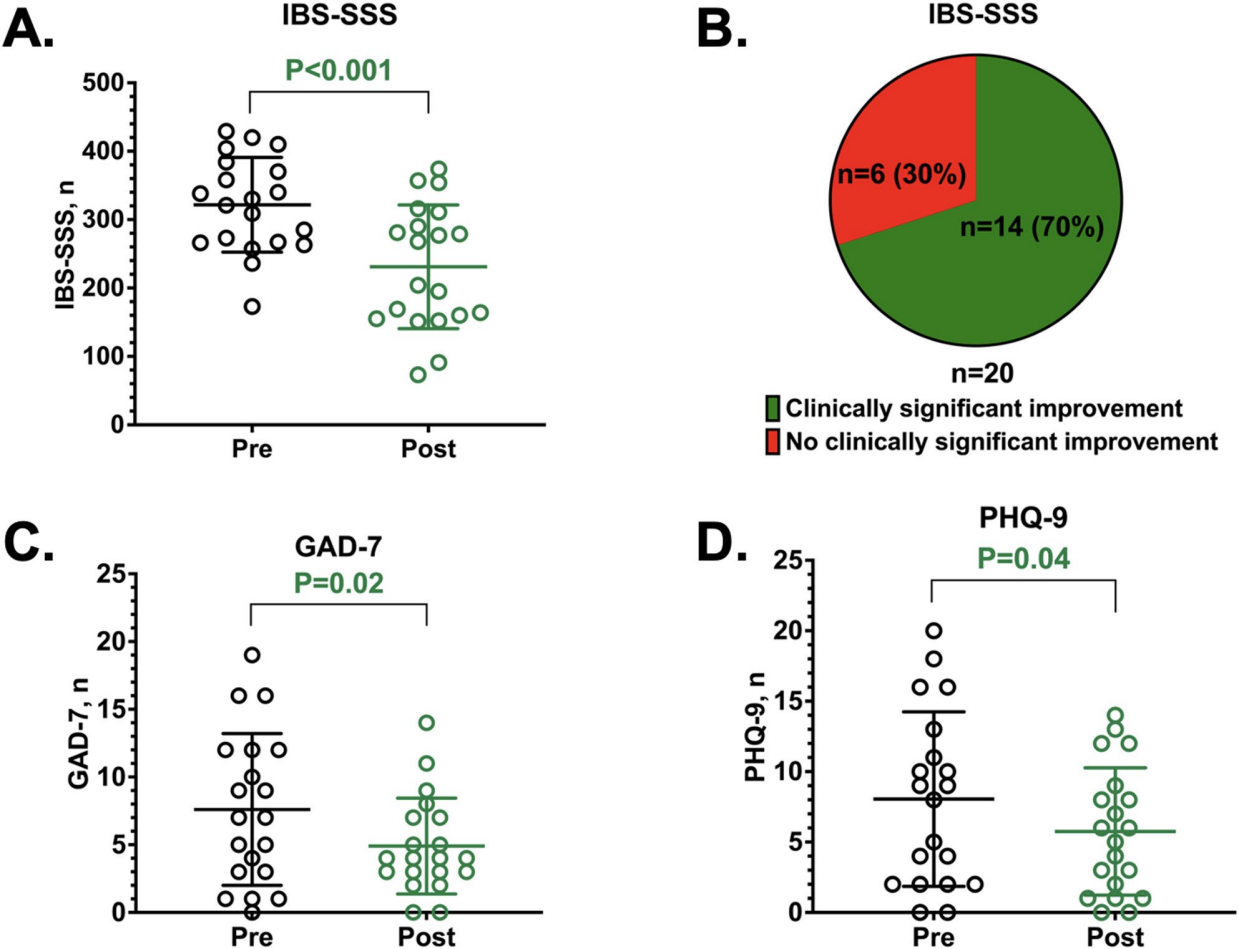
Changes in the IBS-SSS (Panels **A** and **B**), GAD-7 (Panel **C**), and PHQ-9 (Panel **D**) scores. Abbreviations: GAD-7, generalized anxiety disorder-7 scale; IBS-SSS, irritable bowel syndrome symptom severity scale; PHQ-9, patient health questionnaire-9

Among the 20 patients who provided follow-up questionnaire data, the mean baseline IBS-SSS, PHQ-9 and GAD-7 scores were consistent with severe IBS (321.65 [69.21]), mild depression (8.05 [6.19]), and mild anxiety (7.60 [5.61]), respectively **(**Table 2**).** Among the 20 individuals, there was a statistically significant reduction in the IBS-SSS (*P* < 0.001), GAD-7 (*P* = 0.02), and PHQ-9 (*P* = 0.04) scores **(**Fig. 2**).** Fourteen (70.0%) patients reported a clinically significant improvement in the IBS-SSS **(**Fig. 2**).**

**Table 2:** Questionnaire scores at baseline and follow-up

Subgroup Labels	Patients who consented for treatment (*n* = 30)	Patients with two-point data *AND* completed the entire CBT course (*n* = 15)	Patients with two-point data (*n* = 20)	Patients with no follow-up data *OR* partially completed the CBT course (*n* = 15)	Patients with no follow-up questionnaire data (*n* = 10)	Patients with follow-up data who partially completed the CBT course (*n* = 5)	*P* value^a^ A vs. C	*P* value ^a^ A vs. D	*P* value ^a^ A vs. E	*P* value ^a^ B vs. D
		Subgroup A	Subgroup B	Subgroup C	Subgroup D	Subgroup E				
IBS-SSS, mean (SD)	328.77 (80.58)	317.67 (72.86)	321.65 (69.21)	339.87 (88.75)	343.00 (102.32)	333.60 (62.70)	0.35	0.29	0.80	0.31
GAD-7, mean (SD)	7.55 (5.41)	8.80 (5.52)	7.60 (5.61)	6.21 (5.15)	7.44 (5.27)	4.00 (4.58)	0.19	0.56	0.14	0.95
PHQ-9, mean (SD)	9.03 (6.57)	9.20 (6.36)	8.05 (6.19)	8.87 (7.00)	11.00 (7.20)	4.60 (4.56)	0.81	0.57	0.08	0.29
Post IBS-SSS, mean (SD)	N/A^b^	221.07 (93.57)	231.05 (90.39)	N/A^c^	N/A^d^	261.00 (81.68)	N/A	N/A	0.35	N/A
Post GAD-7, mean (SD)	N/A^b^	5.13 (3.44)	4.90 (3.54)	N/A^c^	N/A^d^	4.20 (4.15)	N/A	N/A	0.55	N/A
Post PHQ-9, mean (SD)	N/A^b^	5.87 (4.63)	5.75 (4.52)	N/A^c^	N/A^d^	5.40 (4.67)	N/A	N/A	0.80	N/A
Change in IBS-SSS, mean (SD)	N/A^b^	-96.60 (84.71)	-90.60 (75.47)	N/A^c^	N/A^d^	-72.60 (37.41)	N/A	N/A	0.80	N/A
Change in GAD-7, mean (SD)	N/A^b^	-3.67 (4.25)	-2.70 (4.31)	N/A^c^	N/A^d^	0.20 (3.27)	N/A	N/A	0.07	N/A
Change in PHQ-9, mean (SD)	N/A^b^	-3.33 (3.89)	-2.30 (4.17)	N/A^c^	N/A^d^	0.80 (3.70)	N/A	N/A	0.08	N/A

*Abbreviations*: *CBT* cognitive behavioral therapy, *GAD-7* generalized anxiety disorder-7, *IBS* irritable bowel syndrome, *IBS-SSS* irritable bowel syndrome symptom severity scale, *PHQ-9* patient health questionnaire-9

^a^ Wilcoxon-Rank Sum or Fishers-Exact test for continuous and categorical variables, respectively

^b^ Identical to subgroup B

^c^ Identical to subgroup E

^d^ No follow-up questionnaire data

### Baseline characteristics associated with clinical response

Among the 20 patients with baseline and follow-up data, age was not significantly correlated with symptom improvement in the IBS-SSS (ρ=-0.28, *P* = 0.23), PHQ-9 (ρ=-0.33, *P* = 0.15), nor GAD-7 (ρ=-0.23, *P* = 0.33) (Supplementary Fig. 1). However, the 14 individuals who reported a clinically significant improvement in the IBS-SSS were older (51.00 [13.17] vs. 35.33 [15.36] years, *P* = 0.04) than the six individuals for whom this was not the case (Supplementary Fig. 1).

Symptom improvement was more noticeable among men than women with respect to the IBS-SSS (-136.86 [73.31] vs. -65.69 [66.41], *P* = 0.06) and PHQ-9 (-4.71 [3.50] vs. -1.00 [4.02], *P* = 0.06), but not the GAD-7 (-1.86 [3.48] vs. -3.15 [4.76], *P* = 0.59) (Supplementary Fig. 2). All seven men in the study reported a clinically significant improvement in the IBS-SSS, while this was only identified among 7 of the 13 (53.8%) women (*P* = 0.04) (Supplementary Fig. 2).

A longer interval between IBS symptom onset and prescription of the application was significantly associated with symptom improvement in the PHQ-9 (ρ=-0.55, *P* = 0.04), but not the IBS-SSS (ρ=-0.27, *P* = 0.36) nor the GAD-7 (ρ=-0.08, *P* = 0.78) (Supplementary Fig. 3). 

**Fig. 3:**
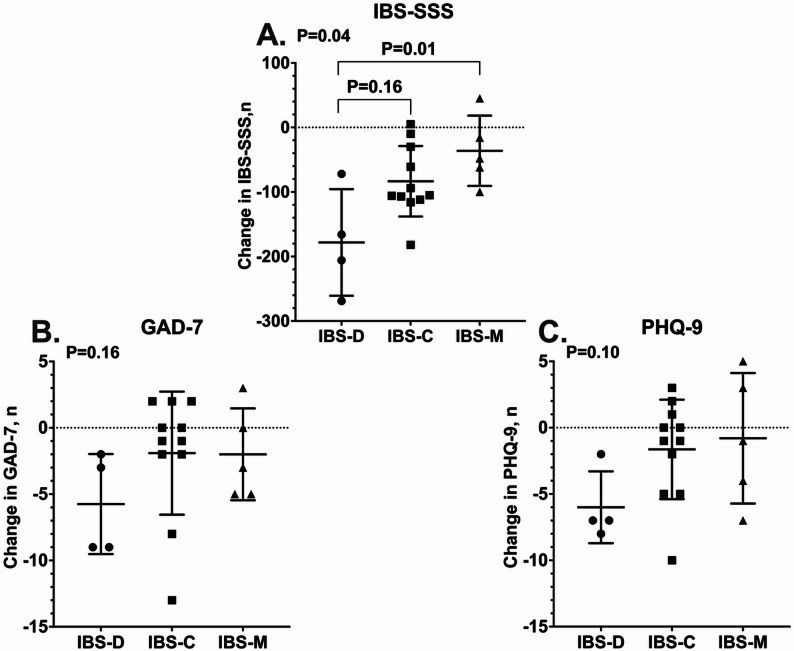
Association between IBS subtype and changes in the IBS-SSS (Panel **A**), GAD-7 (Panel **B**), and PHQ-9 (Panel **C**). Abbreviations: GAD-7, generalized anxiety disorder-7, IBS, irritable bowel syndrome; IBS-SSS, irritable bowel syndrome symptom severity scale, PHQ-9, patient health questionnaire-9

Patients with IBS-D reported the greatest improvement in the IBS-SSS compared to individuals with IBS-C and IBS-M (Fig. 3). Individuals with IBS-D had a significantly greater reduction in the IBS-SSS compared to those with IBS-M (*P* = 0.01), but not IBS-C (*P* = 0.16). The distribution of the PHQ-9 (*P* = 0.10) and GAD-7 (*P* = 0.16) scores were not significantly different between the IBS subtypes (Fig. 3).

### Baseline characteristics associated with dropout

Individuals who completed the entire course had a higher body mass index (BMI) versus drop-outs (*P* = 0.006) **(**Table 1**).** The baseline IBS-SSS (*P* = 0.35), GAD-7 (*P* = 0.19) and PHQ-9 (*P* = 0.19) scores were not significantly different between persons who completed the entire CBT intervention versus drop-outs **(**Table 2**).**

## Discussion

To our knowledge, this is the first publication assessing the efficacy of the Mahana™ IBS CBT application in a real-world setting among patients with IBS recruited in the UK. In this single-center prospective study, 50% of participants who consented to treatment completed a ten-week course of gut-directed CBT and provided questionnaires at baseline and follow-up. Among the 20 patients who completed baseline and follow-up questionnaires, there was a statistically significant reduction in the IBS-SSS, PHQ-9 and GAD-7 scores. There was a signal to suggest that older participants, men, and those with the IBS-D subtype may have derived greater relief in gastrointestinal and psychological symptoms from using the CBT application.

Our observation that older age was associated with symptom improvement aligns with the results from a study of a gut-directed digital hypnotherapy application [32], highlighting the potential efficacy of digitally delivered gut-directed psychological interventions in a population traditionally considered less familiar with mobile technology [33]. Older individuals may respond better to gut-directed psychological therapy because they are more likely to engage in treatment [23, 34–36] and, consequently, derive greater benefit, possibly because they have tried multiple treatments in the past without success [37]. Moreover, it has been suggested that younger individuals may demonstrate a slower response to CBT compared with older adults [38], so a longer follow-up period may have been able to capture further treatment effects.

Females accounted for 66.7% of patients in our sample, which reflects the higher population prevalence of IBS in women versus men [39]. While previous studies evaluating the efficacy of digitally delivered gut-directed CBT for IBS have not studied the prognostic impact of sex [21, 23, 40–46], we observed that men were more likely to have a clinical response to the intervention versus women. This observation contrasts with existing data showing that women report greater commitment to, and stronger belief in, the effectiveness of CBT versus men [47]. It is possible that the disparity in outcomes between sexes in our study reflects differences in computer self-efficacy and digital operational skills [48]. Our findings may also reflect differences in engagement, as male sex has been associated with higher module completion rates in online depression interventions [49]; however, we observed no sex-based differences in course completion rates, which may reflect limited statistical power rather than a true absence of effect.

IBS-C was the most common subtype of IBS reported by patients who consented to treatment. While other studies evaluating psychological therapy in the treatment of IBS have not demonstrated any differences in outcomes between patients with different IBS subtypes [23, 50], we demonstrated that patients with IBS-D tend to report a better clinical response to the intervention. The clinical relevance of this is questionable, since the CBT application did not concentrate on one specific IBS subtype. These results may instead reflect the poorer quality of life reported by patients with IBS-D compared to other subtypes [51], which may in turn motivate greater engagement with treatment.

A longer duration between IBS symptom onset and initiation of the intervention was associated with greater improvement in depressive symptoms, which may reflect increased motivation to engage among patients with longstanding symptoms; however, this association was not observed for anxiety or IBS symptom severity and may represent a type I error given the small sample size.

The mean baseline severity of IBS determined using the IBS-SSS in our sample of patients (328.77) was higher than that observed in the pivotal ACTIB trial (265.0), which primarily recruited patients from primary care [21], and IBSOS (281.9) [52] tertiary care trial. The reduction in the IBS-SSS at week 12 was comparable between this uncontrolled trial, the web-based CBT arm of the ACTIB trial [21], and intention-to-treat group of the minimal therapist contact CBT arm of the IBSOS trial [52]. These findings suggest that digital gut-directed CBT works similarly in different healthcare settings among individuals with a range of IBS symptom severity.

50% of patients (*n* = 15) adhered to the entire study protocol, which is higher than the proportion observed in a real-world US setting of the Mahana™ application (19%) [23]. There were no significant differences in baseline scores or symptom change (IBS-SSS, PHQ-9, or GAD-7) between individuals who completed all ten CBT (*n* = 15) modules and those who partially completed the course (*n* = 5). Individuals with higher BMI were more likely to engage with the CBT application, which could be secondary to the privacy and non-judgmental nature of online platforms, which may help users avoid the weight-related stigma sometimes experienced in face-to-face healthcare settings. In a US setting, adherence to online CBT has been associated with older age, a prescription by a clinician, and payment for usage [23]. In our study the application was recommended by a clinician and patients did not pay for treatment.

Strengths of this study include the use of validated questionnaires and the real-world clinical setting in which it was conducted, which enhances the generalizability of the findings to routine clinical practice. Nevertheless, this study has several limitations. Its uncontrolled design limits causal inference, and reliance on self-reported data may introduce reporting bias. The small sample also increases the risk of type II error for those comparisons where no significant difference was found. Recruitment was halted early following the sponsor’s decision to discontinue development of the application, resulting in reduced statistical power.

Additionally, the sample may not be representative of all IBS patients, particularly those less comfortable with digital health tools, or those consulting in non-tertiary care settings. Finally, the lack of long-term follow-up limits conclusions on the durability of symptom improvement, as follow-up was not included in our ethical approval, although this likely reflects real-world practice where few clinics collect long-term patient-reported outcomes following CBT [53].

Future studies should assess the baseline characteristics of patients who did not consent to treatment and the reasons why, using qualitative methods. The instruments used in this study (IBS-SSS, GAD-7, PHQ-9) are validated as composite measures and are not designed to distinguish between specific domains of change (e.g. cognitive, behavioral, or emotional factors). As such, we were unable to determine whether particular components improved preferentially. Therefore, future studies should incorporate more granular, domain-specific measures to better characterize the pathways through which digital CBT exerts its effects in IBS. Future research should also assess treatment effects over a longer timeframe to confirm the durability of symptom improvement, ideally using symptom diaries, which are arguably more reliable than recall [54]. The Mahana™ IBS CBT application was provided free of charge in this study; in other settings, associated costs may influence uptake, engagement, and adherence, and should be considered in future studies evaluating scalability and implementation. Finally, this study was not powered to assess adverse outcomes, such as psychological deterioration; therefore, wider implementation in future studies should incorporate safety monitoring and clear pathways for clinical escalation, particularly where access to specialist psychological care is limited. 

In conclusion, this study provides preliminary evidence that the Mahana™ IBS CBT application is associated with a statistically significant improvement in gastrointestinal and psychological symptoms in a real-world UK clinical setting.

## Supplementary Information

Supplementary Material 1.

## Data Availability

Data are available from the corresponding author upon reasonable request.

## Abbreviations

BMI: Body mass index
CBT: Cognitive behavioral therapy
DGBI: Disorder of gut-brain interaction
GAD-7: Generalized anxiety disorder-7
IBS: Irritable bowel syndrome
IBS-C: Irritable bowel syndrome-constipation
IBS-D: Irritable bowel syndrome-diarrhea
IBS-M: Irritable bowel syndrome-mixed
IBS-SSS: IBS symptom severity scale
PHQ-9: Patient health questionnaire-9
