# Supplementary material for: Outcomes for a digital prescription mobile application for adults with irritable bowel syndrome: an uncontrolled trial
Source: BMC Gastroenterol. 2026 Jun 11;26:395. doi: 10.1186/s12876-026-04964-6 (PMC13321547; doi:10.1186/s12876-026-04964-6)
Supplement: Supplementary file 1 — Supplementary Material 1. [file 12876_2026_4964_MOESM1_ESM.docx]

**Supplementary Table 1.** The Ten Modules that Compose the Mahana™ IBS Mobile Phone Application. Abbreviations: IBS, irritable bowel syndrome

| **Session number** | **Details** |
| --- | --- |
| Session 1: Symptoms and Stress | - Possible causes of IBS and illustrative physiology of the digestive system together with the functional changes that occur in the gastrointestinal tract because of IBS. The biopsychosocial model of IBS is explained. - How the autonomic nervous system ('fight-or-flight' stress system) can interact with the enteric nervous system. - Self-assessment of the interaction between thoughts, feelings, and behaviours and how these can impact stress levels and gastrointestinal symptoms. - Homework: Symptom diary that collects data on abdominal pain and stress levels, size and time of meals, bowel habits (diarrhoea and constipation), behavioural changes made as a response to IBS. |
| Session 2: Managing Symptoms | - Review of the symptom diary. - Behavioural management of the symptoms of diarrhoea and constipation, and common myths in this area are discussed. Goal setting is explained. - Homework: Goal setting for managing altered bowel patterns. |
| Session 3: Eating Patterns | - The importance of healthy and regular eating patterns is covered. - Homework: Goal setting, monitoring and evaluation continue weekly throughout the programme. Goals around regular and healthy eating are introduced. |
| Session 4: Activity & Exercise Patterns | - Identifying activity patterns such as resting too much in response to symptoms or an all-or-nothing style of activity is addressed. - Importance of exercise and physical movement in symptom management is covered. - Homework: Goal setting for regular exercise and managing unhelpful activity patterns, if relevant. |
| Session 5: Unhelpful Thoughts | - Identifying unhelpful thoughts in relation to IBS symptoms and high expectations of self is introduced. - Link between these thoughts, feelings, behaviours and symptoms is reinforced. - Homework: Goal setting plus daily thought records of unhelpful thoughts. |
| Session 6: Alternative Thoughts | - The steps for coming up with alternatives to unhelpful thoughts are covered together with personal examples. - Homework: Goal setting plus daily thought records including coming up with realistic alternative thoughts. |
| Session 7: Relaxation Techniques | - Diaphragmatic breathing, progressive muscle relaxation and guided imagery relaxation are presented in video and audio formats. - Homework: Goal setting for relaxation techniques. |
| Session 8: Managing Sleep & Stress | - Basic stress management and sleep hygiene are discussed. - Homework: Goal setting for stress management and good sleep habits. |
| Session 9: Managing Emotions | - Users learn how to manage difficult emotions and embrace the experience of pleasant emotions. - Three key steps for managing emotions are covered: Identifying emotions; Accepting emotions; Learning how to manage difficult emotions. - Homework: Emotion record. |
| Session 10: Managing Flare-ups and the Future | - The probability of flare-ups is discussed and users are encouraged to develop achievable, long-term goals and to continue to employ the skills they have learnt throughout the manual to manage flare-ups and ongoing symptoms. |

**Supplementary Figure 1.** Association Between Age and Changes in the IBS-SSS (Panels A and B), GAD-7 (Panel C), and PHQ-9 (Panel D) scores. Abbreviations: IBS-SSS, irritable bowel syndrome symptom severity scale; GAD-7, generalised anxiety disorder-7 scale; PHQ-9, patient health questionnaire 9.

**
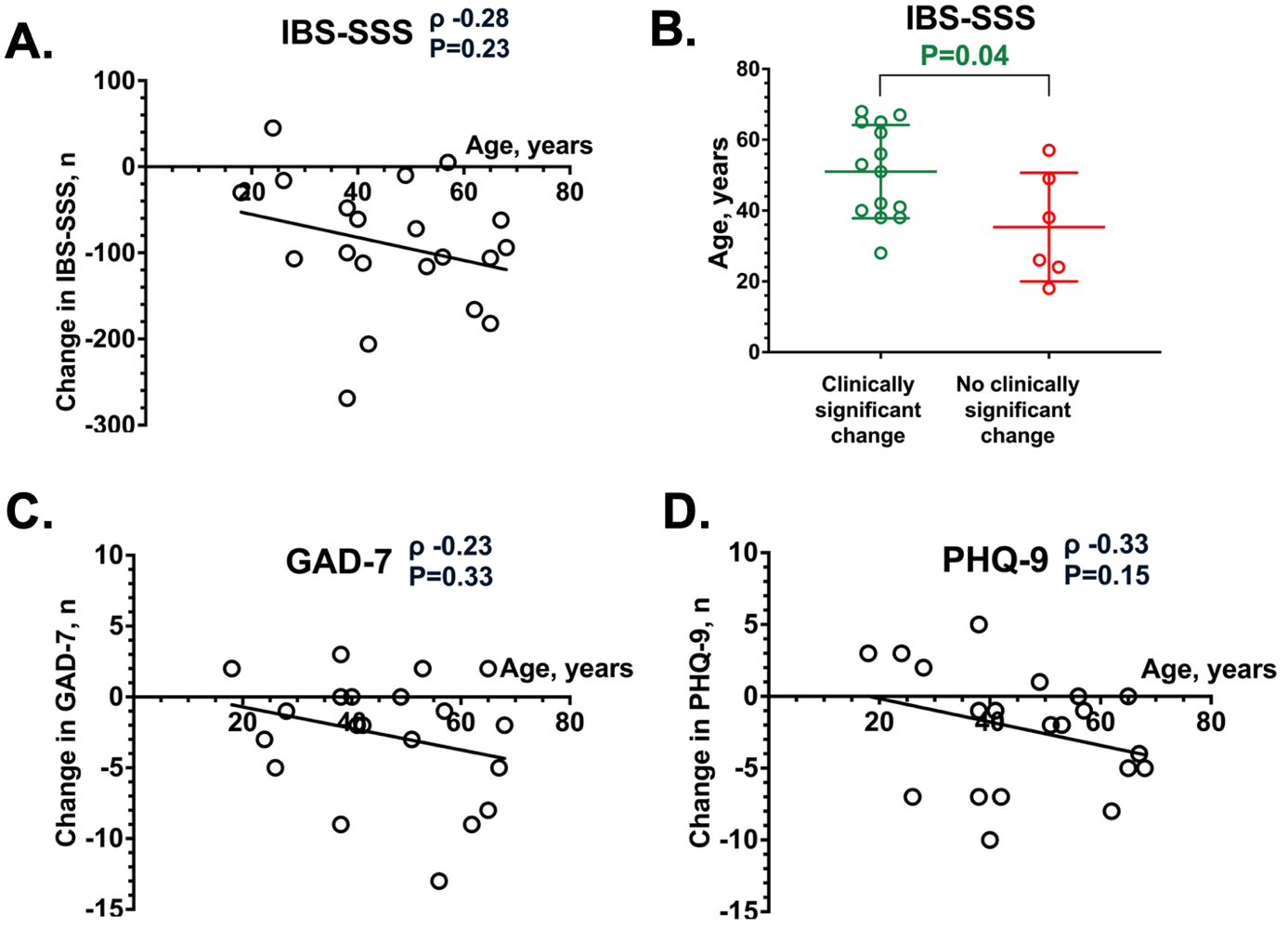
**

**Supplementary Figure 2.** Association Between Sex and Changes in the IBS-SSS (Panels A and B), GAD-7 (Panel C), and PHQ-9 (Panel D) scores. Abbreviations: IBS-SSS, irritable bowel syndrome symptom severity scale; GAD-7, generalised anxiety disorder-7 scale; PHQ-9, patient health questionnaire 9.

**
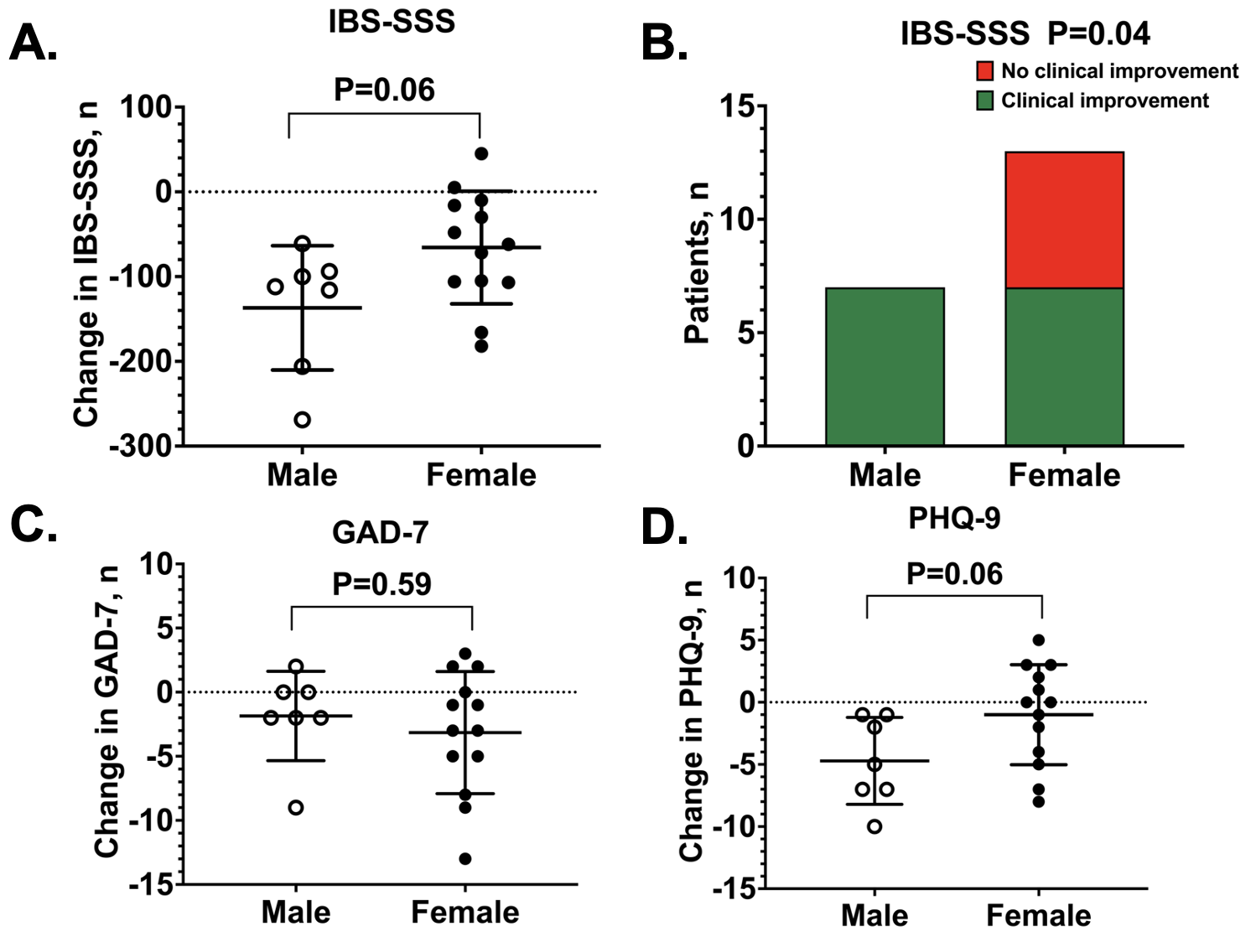
**

**Supplementary Figure 3.** Association Between Time From Symptom Onset to Study Date and Changes in IBS-SSS (Panels A and B), GAD-7 (Panel C), and PHQ-9 (Panel D) Scores. Abbreviations: IBS-SSS, irritable bowel syndrome symptom severity scale; GAD-7, generalised anxiety disorder-7 scale; PHQ-9, patient health questionnaire 9.

**
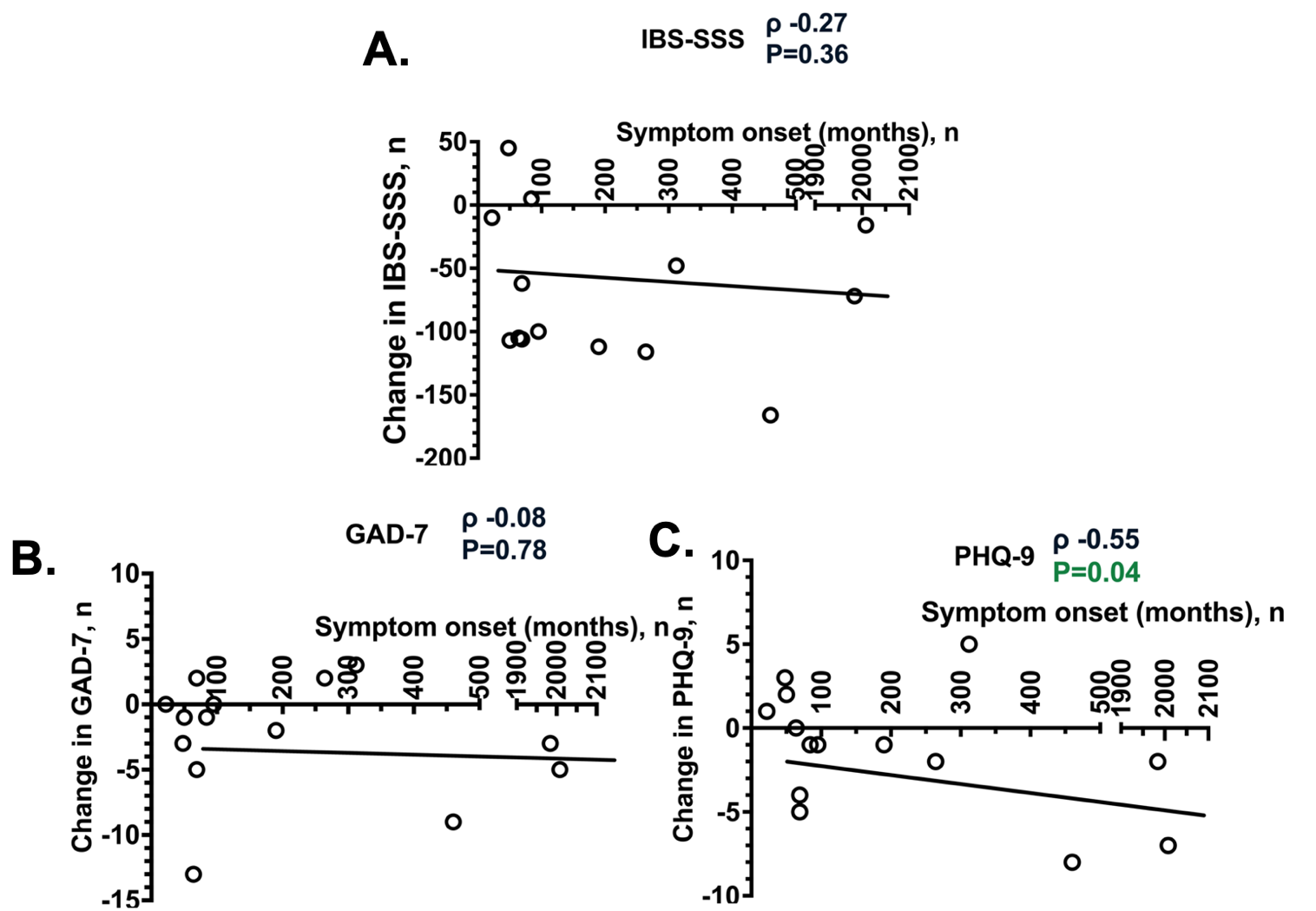
**
